# Supplementary material for: Validity and Effects of Placement of Velocity-Based Training Devices
Source: Sports (Basel). 2021 Aug 31;9(9):123. doi: 10.3390/sports9090123 (PMC8472848; doi:10.3390/sports9090123)
Supplement: Supplementary file 1 [file sports-09-00123-s001.zip › sports-1225326-supplementary/Figure S1.pdf]

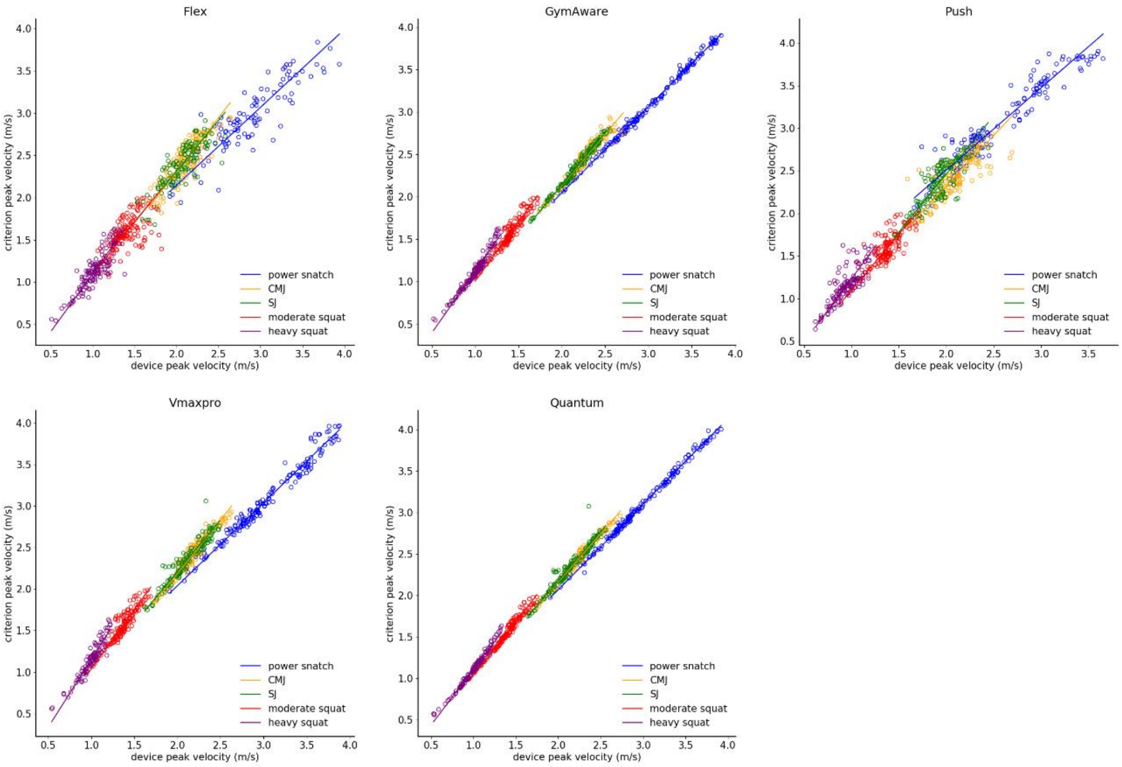

Figure S1. Regression lines for the individual devices (panels) and exercises (colors) for the parameter v_{peak}.

Sports **2021**, *9*, 123. https://doi.org/10.3390/sports9090123 www.mdpi.com/journal/sports
