# Supplementary material for: Validity and Effects of Placement of Velocity-Based Training Devices
Source: Sports (Basel). 2021 Aug 31;9(9):123. doi: 10.3390/sports9090123 (PMC8472848; doi:10.3390/sports9090123)
Supplement: Supplementary file 1 [file sports-09-00123-s001.zip › sports-1225326-supplementary/Figure S4.pdf]

sports

MDPI

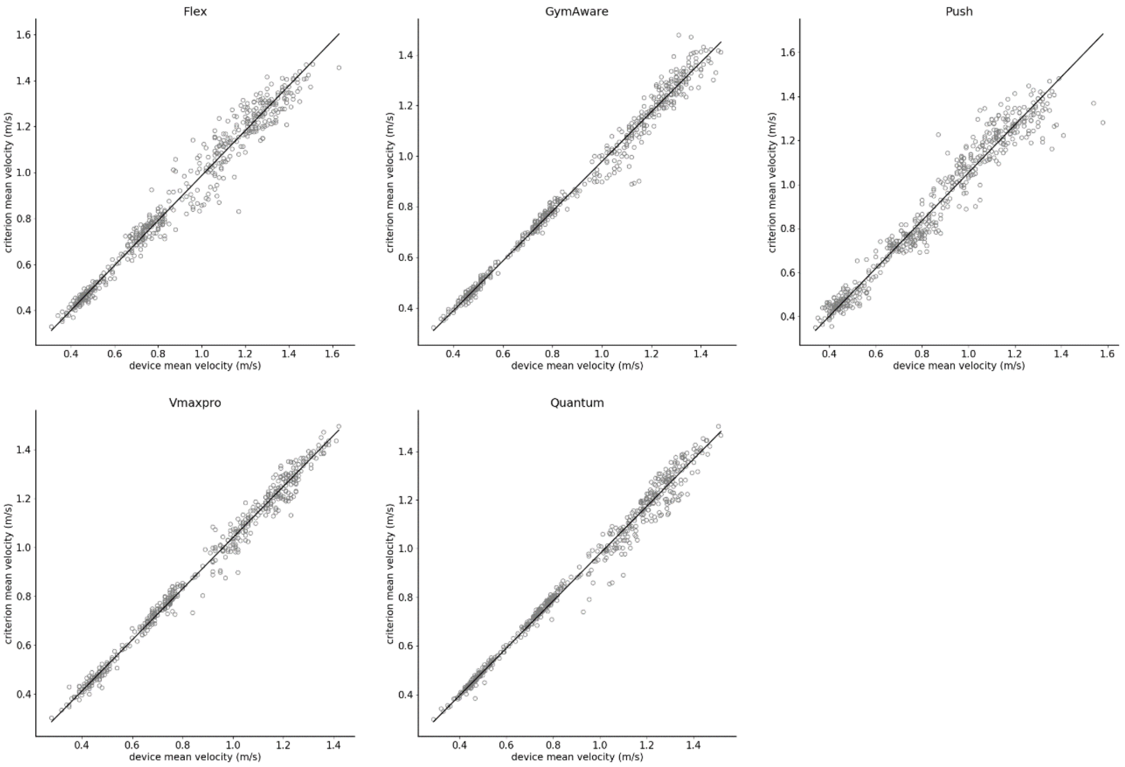

Figure S4. Data point scatter and least-products regression lines for the individual devices (panels) and all exercises pooled together, excluding power snatch, for the parameter v_{mean}. Regressions were generated without power snatch because there appeared to be a systematic difference in v_{mean} between devices and criterion measures.
