# Supplementary material for: Validity and Effects of Placement of Velocity-Based Training Devices
Source: Sports (Basel). 2021 Aug 31;9(9):123. doi: 10.3390/sports9090123 (PMC8472848; doi:10.3390/sports9090123)
Supplement: Supplementary file 1 [file sports-09-00123-s001.zip › sports-1225326-supplementary/Figure S5.pdf]

sports

MDPI

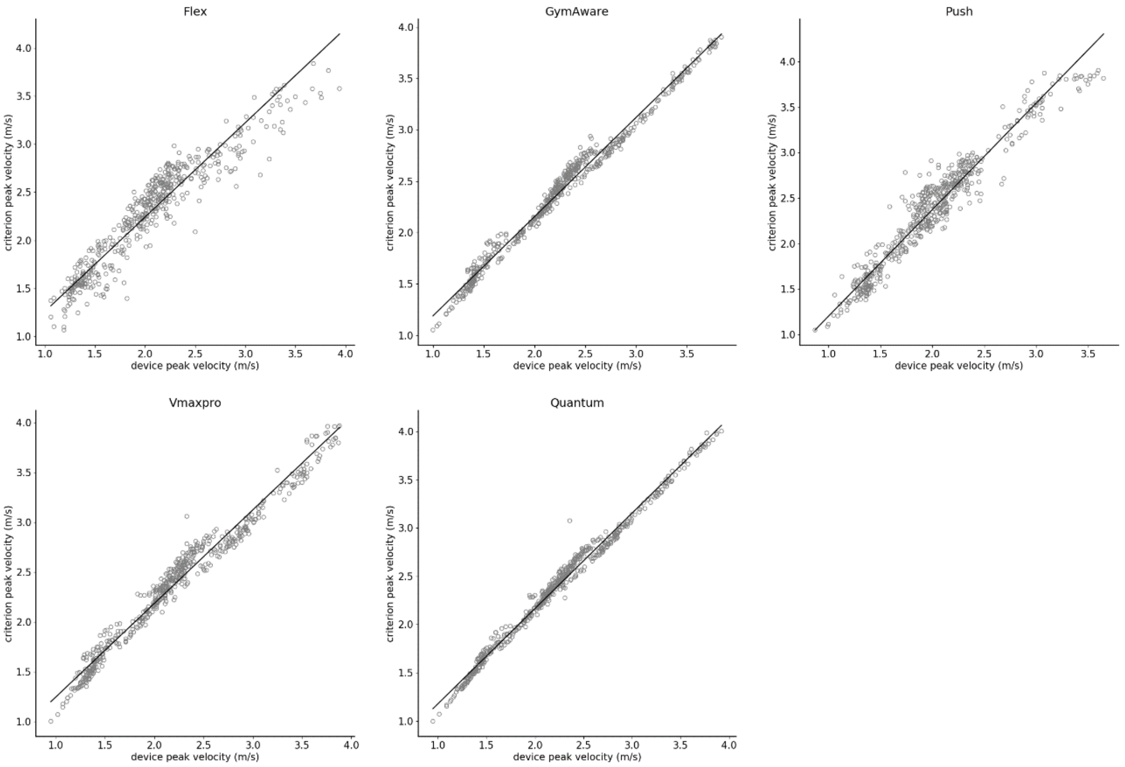

Figure S5. Data point scatter and least-products regression lines for the individual devices (panels) and all exercises pooled together for the parameter v_{peak}.
