# Supplementary material for: Validity and Effects of Placement of Velocity-Based Training Devices
Source: Sports (Basel). 2021 Aug 31;9(9):123. doi: 10.3390/sports9090123 (PMC8472848; doi:10.3390/sports9090123)
Supplement: Supplementary file 1 [file sports-09-00123-s001.zip › sports-1225326-supplementary/Supplementary S1.pdf]

Supplementary S1: Detailed description of motion capture data processing steps

Six reflective markers were placed on each end of the barbell (either on the Flex device or on a clamp of similar diameter) in a symmetrical pattern. After labelling, gaps were filled with one of the filling functions built into Vicon Nexus. Next, all trajectories were smoothed within Nexus using the built-in Woltring-Filter (Filter mode = GCV; Smoothing = 20). Thereafter, marker data were processed using a custom Python script. The main steps in this script were as follows:

1. The positions and trajectories of both barbell ends were identified as the mean of the six markers or (if fewer than six were visible) of two juxtaposed markers.
2. Along the virtual line connecting the two bar ends and based on the measured distance of each device's attachment point from one of the bar ends, a virtual marker along with its trajectory was generated at that point. From these, a three-dimensional velocity signal for the attachment point of each device was calculated as the first derivative of the three-dimensional position signal with the *GetSplineResult* function of the *ViconNexus* Python module.
3. For each repetition and attachment point, the onset and end of the concentric phase was identified as follows:
    a. The onset of the concentric phase was defined as the last frame before the attachment point's vertical velocity became positive (threshold=0 m/s). For the exercise power snatch, because the automatic detection of the concentric phase onset was more difficult, a slightly more conservative vertical velocity threshold of 0.05 m/s combined with a vertical acceleration of 5 m/s^2 was used instead.
    b. The end of the concentric phase was defined as the first frame after phase onset in which vertical velocity dropped below 0 m/s.
4. For each repetition and attachment point, v_{mean} and v_{peak} were extracted as the mean and maximal resultant (scalar) velocity, respectively, within the concentric phase.
