# Supplementary figures and images for: Validity and Effects of Placement of Velocity-Based Training Devices
Source: Sports (Basel). 2021 Aug 31;9(9):123. doi: 10.3390/sports9090123 (PMC8472848; doi:10.3390/sports9090123)

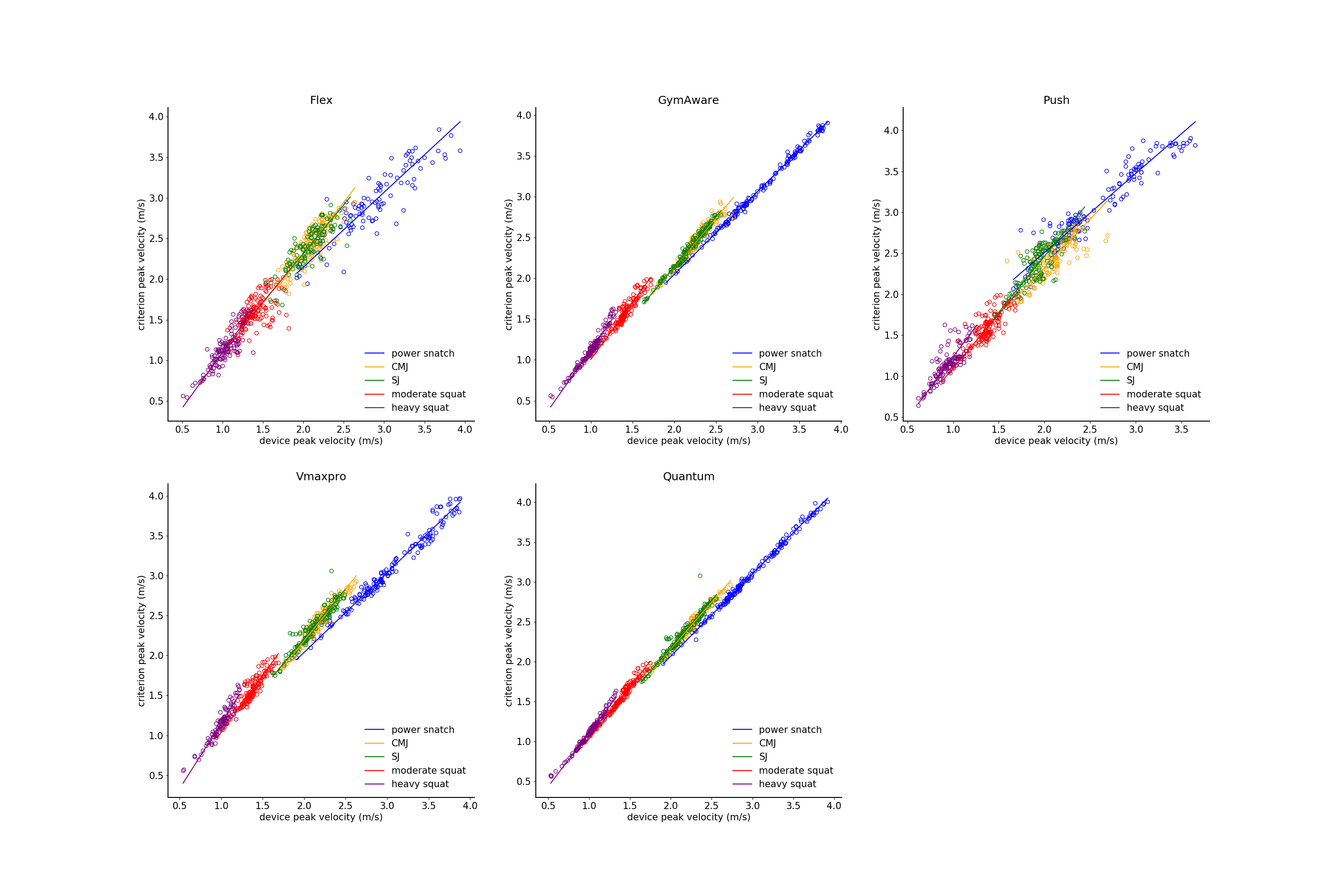

Supplement: Supplementary file 1 [file sports-09-00123-s001.zip › sports-1225326-supplementary/Figure S1.png]

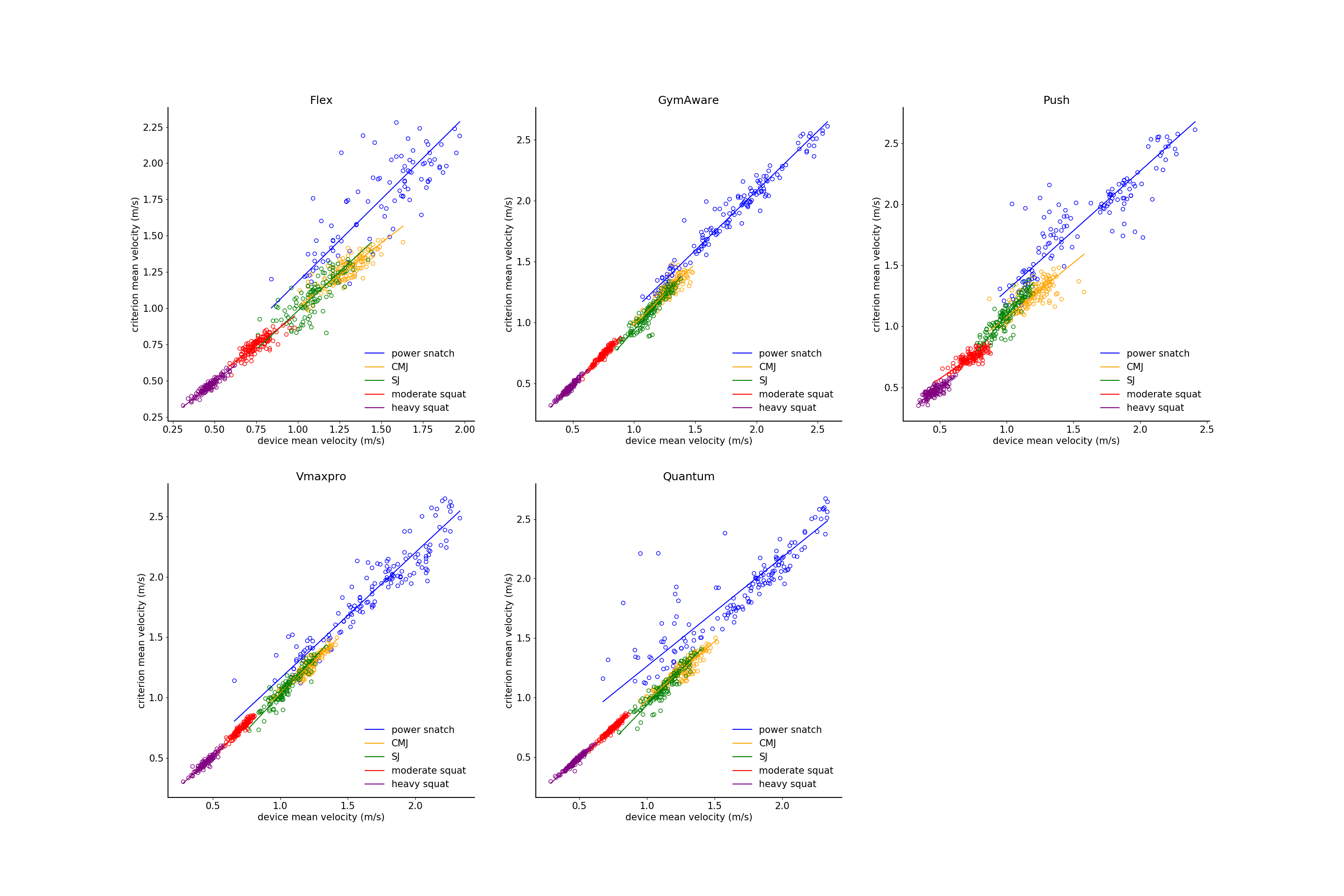

Supplement: Supplementary file 1 [file sports-09-00123-s001.zip › sports-1225326-supplementary/Figure S2.png]

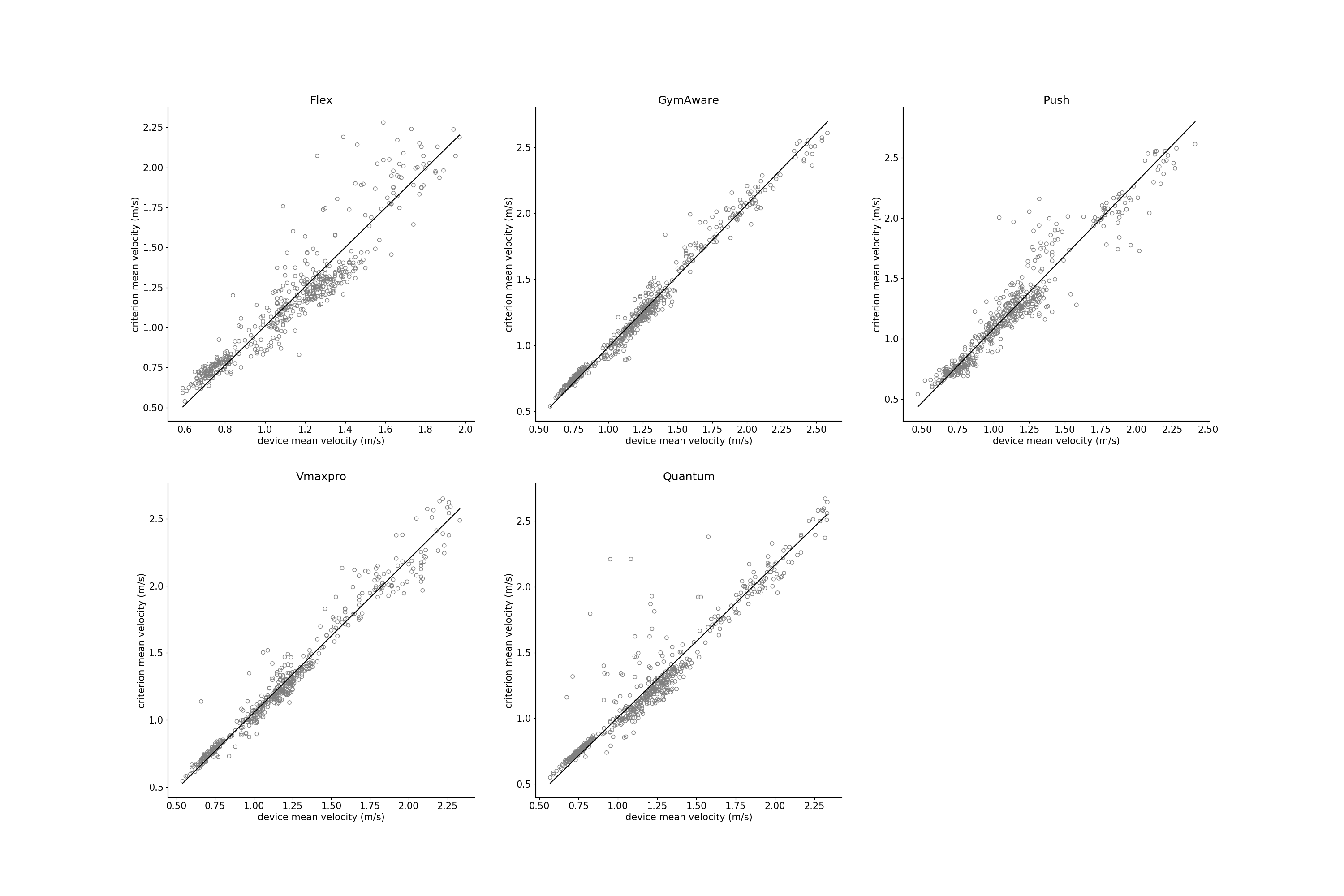

Supplement: Supplementary file 1 [file sports-09-00123-s001.zip › sports-1225326-supplementary/Figure S3.png]

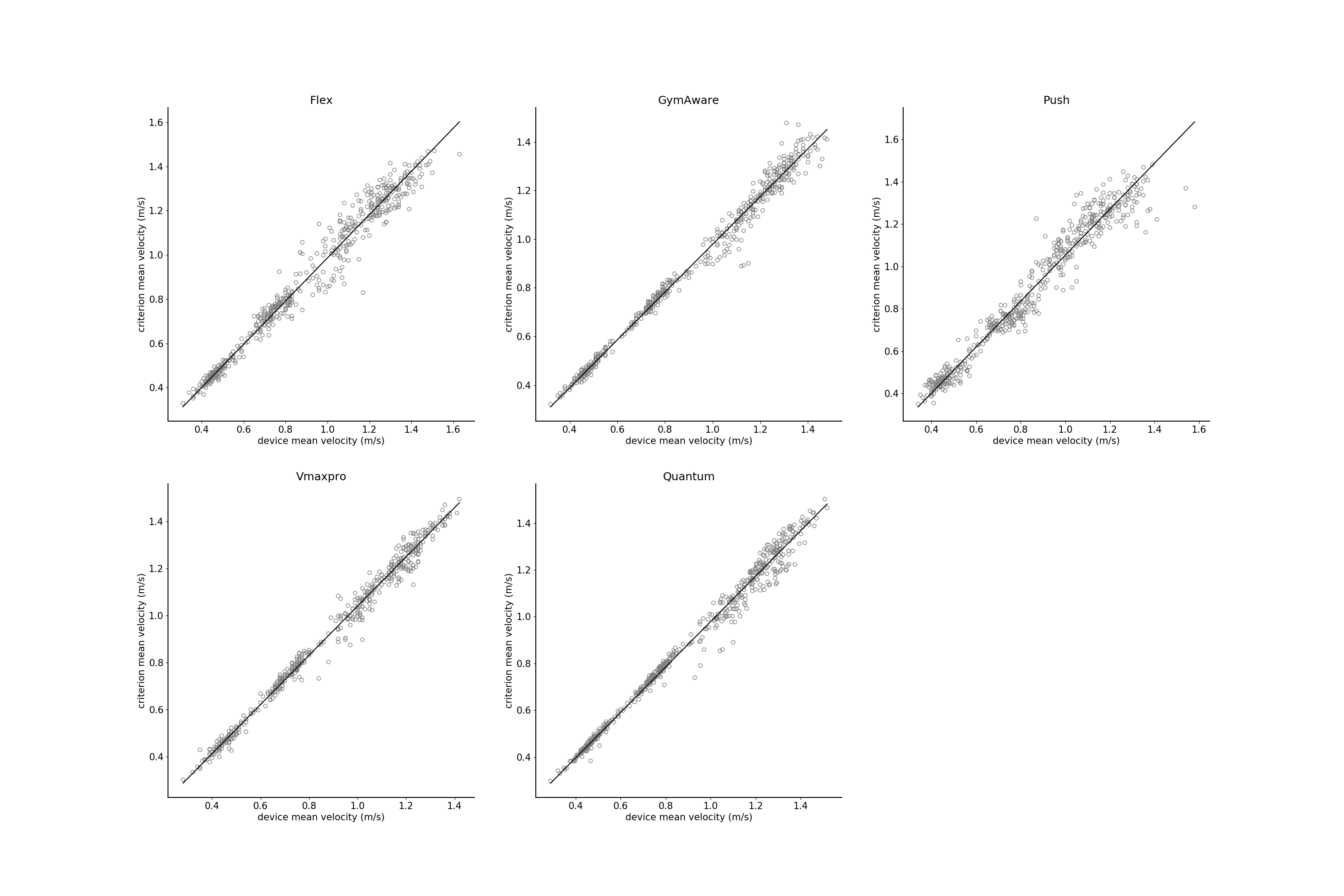

Supplement: Supplementary file 1 [file sports-09-00123-s001.zip › sports-1225326-supplementary/Figure S4.png]

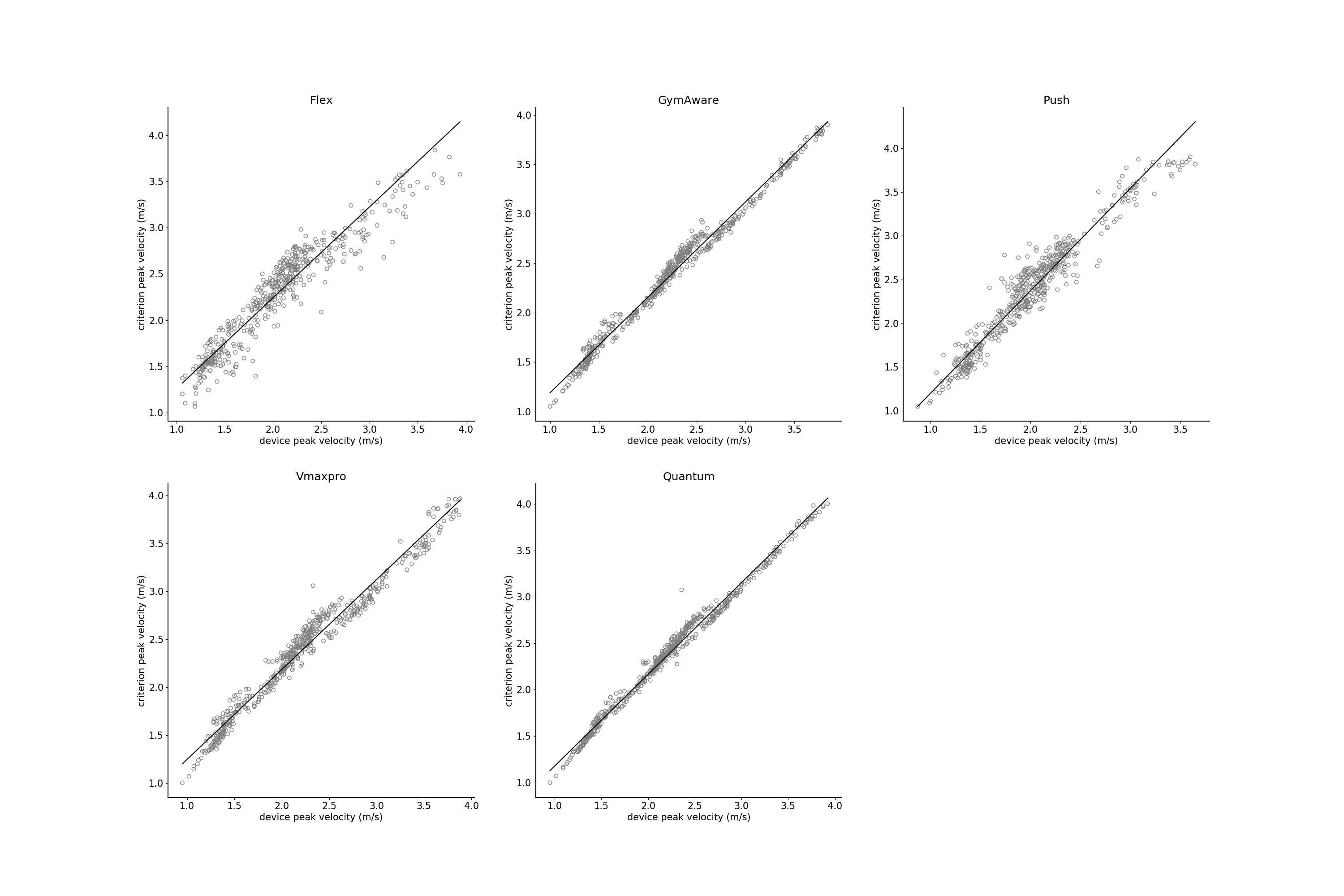

Supplement: Supplementary file 1 [file sports-09-00123-s001.zip › sports-1225326-supplementary/Figure S5.png]
